# Investigating the microwave degradation of polypropylene microplastics and their impact on human intestinal cell models

**DOI:** 10.1016/j.toxrep.2026.102285

**Published:** 2026-05-29

**Authors:** Raphaela O.G. Ferreira, Emine Merve Canga, Aoife Gowen, Tara McMorrow, Jun-Li Xu

**Affiliations:** a UCD School of Biosystems & Food Engineering, University College Dublin, Belfield, Dublin, Ireland; b UCD Conway Institute, University College Dublin, Belfield, Dublin, Ireland

**Keywords:** Polypropylene, Microplastics, Microwave Degradation, Caco-2 cells, O-PTIR

## Abstract

Humans are inevitably exposed to microplastics (MPs) through various pathways, with ingestion being a primary route. Polypropylene (PP), commonly used for food storage and microwave (MW) heating, has the potential to release MPs when exposed to heat or prolonged use. Despite their detection in human tissues, including the gut, the health impacts of degraded PP particles remain poorly understood. This study investigates the characteristics of MW-treated PP particles and their effects on human intestinal Caco-2 cells, focusing on cell metabolic activity, membrane damage, and oxidative stress. Caco-2 cells were exposed to PP-MPs with a concentration of 200 µg/mL subjected to various MW degradation cycles: 3-minute cycles (repeated 1, 5, and 10 times) and a 30-minute continuous cycle at high power (1000 W), for 24 and 48 h. Surface charge and infrared spectral analyses were employed for chemical characterization of the MPs. Toxicological assays were used to evaluate cell metabolism, membrane integrity, and oxidative stress. Results indicated that prolonged MW exposure led to oxidative degradation in the PP-MPs. After 24 h of exposure to MW-treated PP-MPs, an increase in the metabolic activity of Caco-2 cells ranging from 14% to 35% was observed. However, after 48 h, a statistically significant decrease in metabolic activity, ranging from 10% to 14% was observed for the cells treated with PP-MPs subjected to short MW cycles, while a persistent upregulation was observed for the 30 min continuous MW MPs. No membrane damage was detected in Caco-2 cells under any experimental condition. In contrast, oxidative stress (OS) levels surged by at least 74% in all MW cycle treatments after 24 h exposure and remained elevated compared to untreated controls after 48 h, suggesting OS as a key mechanism influencing cytotoxicity. Cell imaging suggested that MW-degraded PP-MPs induced a ROS-driven intracellular vacuolization consistent with early apoptotic signaling in Caco-2 cells. This study enhances our understanding of the biological effects of PP-MPs exposed to MW degradation, highlighting the need for further research into the broader health implications of plastic usage.

## 1 Introduction

Polypropylene (PP) is one of the most extensively produced polymers in Europe [1] and is widely used in industrial applications, particularly food packaging. Its high melting point makes it a preferred material for microwavable containers. However, during microwave (MW) exposure - a common domestic heating method - PP undergoes thermal and oxidative degradation [2], leading to the formation of microplastic (MP) fragments (≤5 mm) [3]. Concerns have arisen about the migration of MPs from PP containers into food during MW heating, prolonged storage, and repeated use [4], [5]. Du et al. [6] investigated PP particle release in Hangzhou and Xiamen after 1 min of MW heating: in Hangzhou the mean particle size was 72 µm with an abundance of 27 ± 13 items per container, while in Xiamen the mean size was 80 µm with 15 ± 6 items per container. Jin et al. [7] confirmed PP particle release under MW conditions of 700 W for 30 s to 3 min, reporting PP-MPs > 10 µm in the range 0.5 × 10^5^–4 × 10^5^ particles cm^2^. He et al. [8] reported PP-MPs migration after 10 min at 700 W, with particle sizes between 5 and 50 µm and concentrations of 38–235 × 10^3^ particles/L.

Once released from food containers, PP-MPs enter the human body primarily through ingestion of contaminated food [9]. After crossing the gastrointestinal lumen via various endocytic pathways, PP-MPs have been detected in multiple human tissues: intervertebral discs, bone, and cartilage [10], lung parenchyma [11], across the upper and lower gut including stomach [12] and colon [13], [14]. To investigate PP-MP cytotoxic effects under controlled conditions, researchers employ the human colon adenocarcinoma (Caco−2) cell line, an *in vitro* model that mimics key features of the intestinal epithelial barrier.

The numerous degradation pathways that MPs undergo due to their ubiquity, alters their morphology by creating cracks, bulges, wrinkles, increased roughness and pores - and chemistry by introducing new functional groups and shifting thermal stability [15]. Therefore, a detailed characterization of key parameters such as particle shape, size [16], zeta (ζ) potential [17], and polymer composition [18], is essential for understanding their environmental fate and transport. To achieve this, researchers employ a suite of methods, including thermochemical identification, standard chemical analyses, and spectroscopic techniques [16]. In the last decade, Optical Photothermal Infrared (O-PTIR) spectroscopy has emerged as a promising technique, offering high spatial resolution at the submicron scale in mid-infrared wavelengths and spectra closely correlated with traditional FTIR (Fourier-Transform Infrared spectroscopy) methods [19]. To enhance O-PTIR’s efficiency in detecting MP degradation, chemometric multivariate techniques such as principal component analysis (PCA) are valuable. PCA condenses complex spectral datasets into principal components (PCs) that capture key variations, significantly simplifying data analysis while retaining essential information [20].

Research on aged MPs and their health effects is limited, comprising only about 10% of studies and with PP among the least investigated polymers [21]. As an example, Roursgaard et al. [22] mechanically degraded commercial food containers to isolate PP nanoparticles (NPs), reporting a mean diameter of 158 nm and concentrations in the range 0.003–0.175 µg·mL⁻¹ . Xia and Wang [23] simulated real-life thawing and reheating by applying MW degradation to PP containers, obtaining particles with a size distribution from 1 nm to 5 µm and concentrations between 5 and 100 µg·mL⁻¹ . Jin et al. [7] combined hydrothermal leaching in hot water or food simulants with microwave treatment to extract PP-MPs. Despite these extraction and characterization efforts, the effects of MW-degraded PP on cellular health remain insufficiently explored. To narrow this gap, our study aimed to investigate the effects of MW degradation on PP-MPs through O-PTIR analysis for extensive characterization and evaluate their impacts on Caco−2 cells regarding metabolic activity, membrane damage, and oxidative stress.

## 2 Materials and methods

### 2.1 Polypropylene microwave degradation

Spherical-shaped MPs are frequently used in many MP studies, but these particles do not accurately reflect real MPs present in the environment [24]. Thus, we utilized irregularly shaped PP-MPs in this study to reflect real world conditions. White irregular shaped PP fragments, with a density of 0.90–0.91 g/cm³ and a typical particle size of 55–75 µm, were obtained from Goonvean Fibers Ltd. (Catalogue Number HM20/70 P, Cambridge, United Kingdom). Ten milligrams of these particles were evenly spread on the base of a glass Petri dish (5 cm in diameter), which had been sterilized with 70% ethanol to minimize dust and plastic contamination. The dish was then placed in the middle of a rotating turntable of a Microwave (Model NN-DS59, Panasonic) operating at 1000 W and 2450 MHz. The MW-degradation protocol consisted of 3-minute cycles, repeated 1, 5, or 10 times, and a separate 30-minute continuous cycle. In between each cycle, the samples were allowed to cool to room temperature.

### 2.2 PP-MP characterization and dose estimation

#### 2.2.1 Chemical and morphological characterization

The initial characterization of pristine PP-MPs was performed as follows: 100 µg/mL (w/v) PP solution was prepared in pure water (water type I, 18.2 MΩ.cm, Thermo Scientific Barnstead Smart2Pure water purification system, USA). Following MW heating, 5 mg of each MW-treated PP sample was suspended in pure water to obtain the same concentration (100 µg/mL, w/v). 200 µL aliquots of each dispersion were placed onto regular glass microscope slides (clear glass, 76 ×26 mm, Academy, UK) and allowed to dry under partial cover with glass Petri dishes in a fume hood (WS(G)−71 1800 mm STO VAV) at room temperature (20°C) to prevent air contamination. Chemical characterization of pristine and MW-treated PP-MPs was achieved through O-PTIR spectroscopy obtaining IR spectra in the mid-IR range (1801–769 cm^−1^) and optical images. Analysis was conducted on at least 50 particles with three spectra for each MP. Particles were selected from distinct regions at the centre of each dried droplet on the glass slides to ensure even distribution and to minimize interference from aggregation caused by the coffee-ring effect. Three glass slides were prepared as replicates for each MP type, either pristine or MW-treated samples. In an additional experiment, pristine PP-MPs on glass slides were subjected to repeated MW treatment, enabling the same particles to be re-examined after heating. The same protocol was also applied for the 30-minute MW heating.

Spectral pre-processing involved moving median for noise reduction (a window size of 11), standard normal variate (SNV) to correct for scattering, and polynomial baseline correction. Conversely, baseline correction was intentionally excluded to maintain inherent spectral variance for principal component analysis (PCA), with only moving median filtering and SNV applied. The IR spectra of the samples were then analyzed using PCA to evaluate variation between pristine and MW-treated PP-MPs. The relative weights of specific wavelengths in comparison to each other and their contribution to specific PCs were investigated by inspection of PC loadings.

The morphological characterization of PP Pristine and MW-treated samples proceeded using 10 × microscope images obtained from O-PTIR instrument via MATLAB (R2024b, The MathWorks, Inc., Natick, MA, USA) incorporating functions from the Image Processing Toolbox and Statistics and Machine Learning Toolbox. Equivalent diameter and circularity (%) values were calculated by masking the particles with ‘regionprops’ function of MATLAB. A paired *t*-test was performed to determine the statistical difference (p < 0.05) between the morphological characteristics of pristine and MW-treated samples.

#### 2.2.2 Zeta (ζ) potential measurement

For Zeta (ζ) potential analysis, homogeneous dispersions of pristine and MW-treated PP-MPs were achieved using a non-ionic surfactant, 0.1% Triton X−100 (v/v), as the solvent [25]. ζ-potential values were then measured using the Zetasizer Nano ZS instrument (Malvern Panalytical Ltd., Malvern, UK) with folded capillary cells (DTS1070). The refractive indexes of the solvent and PP-MPs were set at 1.33 and 1.47, respectively and ζ-potential was calculated based on the Smoluchowski model. The ζ-potential measurement was performed in triplicate, with each replicate representing the average of ten individual measurements. Statistical analysis was performed using GraphPad Prism Software Version 8.0 (San Diego, CA, USA) using one-way (ANOVA) for comparisons of multiple groups with significance levels set at (p) < 0.05.

#### 2.2.3 Dose estimation

The approximate number of PP particles present at each nominal concentration was estimated using geometric and density-based calculations. Assuming spherical geometry with a mean diameter of 30 µm (radius 15 µm = 1.5 × 10⁻³ cm) and a PP density of 0.90 g/cm³ , the particle volume was calculated using V=$\frac{4}{3}$πr^3^, yielding 1.41 × 10^−8^ cm³ . Multiplying by the density of PP gives a mean particle mass of 1.27 × 10^−8^ g (0.0127 µg). The number of particles per mL was obtained by dividing the nominal concentration (µg/mL) by the mass per particle, resulting in ∼3.9 × 10 ³ particles/mL at 50 µg/mL, ∼1.6 × 10⁴ particles/mL at 200 µg/mL, and ∼3.9 × 10⁴ particles/mL at 500 µg/mL. In the 96-well format used here (100 µL per well), this corresponds to approximately 3.9 × 10², 1.6 × 10 ³ , and 3.9 × 10 ³ particles per well, respectively. Because the microwave-generated PP particles exhibit irregular morphology and variable aspect ratios, these values represent upper-bound estimates; irregular fragments typically have greater mass per particle than spheres of equivalent diameter, meaning the true particle count per unit mass is likely lower. These estimates are therefore provided as complementary metrics alongside nominal mass concentrations.

### 2.3 Cytotoxicity assays

#### 2.3.1 Caco−2 cell line protocol

Human colorectal adenocarcinoma Caco−2 cells (passages 10–20, ATCC HTB−37) were used to measure the cellular toxicity of PP-MPs before and after MW aging. The cells were cultured in Dulbecco's modified Eagle medium containing 4.5 g/L glucose (DMEM, Merck), supplemented with 10% fetal bovine serum (FBS, Merck), 1% L-glutamine (Gibco, Merck), 1% non-essential amino acids (ThermoFisher Scientific), 100 U/mL penicillin and 100 μg/mL streptomycin (Gibco, Merck). Cell cultures were maintained in cell culture flasks (75 cm^2^ cell culture area; CellStar, Greiner) in a humidified incubator with 5% CO_2_ at 37 °C. When the cell density reached 80–90%, Caco−2 cells were isolated using 0.25% trypsin-EDTA (Gibco, Merck). For assessment of cell proliferation, Caco−2 cells were counted using a hemocytometer. Cell cultures were routinely tested for mycoplasma contamination and found to be negative.

#### 2.3.2 Dose-response study set up

To select exposure concentrations for the MW degradation experiments, pristine PP‑MPs were evaluated in an initial dose-response study. Based on preliminary in‑house data [26], PP powder was prepared to yield final nominal concentrations of 50, 200 and 500 µg/mL in complete DMEM. Dispersions were prepared fresh for each experiment and sonicated for 30 min immediately prior to addition to the well plates. Final concentration selection for the MW degradation toxicological assays was guided by a cytotoxicity cutoff (≤ 20% loss in metabolic activity at 48 h to retain measurable biological signal.

#### 2.3.3 MW PP-MPs study set up

For the MW degradation experiment, PP powder was weighed to produce a concentration of 200 μg/mL and degraded as previously described in Section 2.1. Samples were freshly prepared for each run and subjected to 30 min of sonication prior to addition to the well plates.

#### 2.3.4 MTT (3-(4,5-dimethylthiazol−2-yl)−2,5-diphenyltetrazolium bromide) assay

Caco−2 cells were seeded in 96-well plates at a density of 2 × 10^4^ cells/well in 100 μL culture medium and incubated overnight to allow adhesion. After 24 h, the medium was replaced with 100 μL of the PP-MPs solution (dose–response and MW degradation) and cells were incubated for 24 or 48 h. Controls were run in parallel: untreated control - cells in fully supplemented DMEM; negative control - cells in fully supplemented DMEM with 10% DMSO (cytotoxic control). In addition, media-only blanks (DMEM with no cells) and particle-only blanks (DMEM containing PP particles but no cells) were included to assess background absorbance and any interference of particles with the MTT readout. Well positions were organized to minimize edge effects.

Cell Proliferation Kit I (MTT) was used according to the manufacturer’s instructions (Roche). MTT reagent (final concentration 0.5 mg/mL) was added and incubated for 4 h at 37°C and then 100 μL of solubilization buffer was added for overnight incubation. Absorbance was measured at 550 nm (reference at 690 nm) using a microplate reader (FLUOstar omega, BMG Labtech, Germany). The relative cell metabolic activity (%) was calculated using the 550 nm values as a ratio of experimental groups to the untreated group. The absorbance obtained strongly correlates with the number of metabolic active cells [27]. Although the MTT assay is predominantly associated with mitochondrial activity or cell viability [28], [29], mounting evidence indicates that formazan formation also involves the plasma membrane, lipid droplets, endosomes, and lysosomes [30], [31]. Thus, our MTT results should be interpreted as reflecting overall cellular metabolic activity [32].

#### 2.3.5 Lactate dehydrogenase (LDH) release assay

Cell membrane damage was assessed using the Invitrogen™ CyQUANT™ LDH Cytotoxicity Assay Kit. Three technical replicates of Caco−2 cells were seeded into a 96-well-plate (2 × 10^4^ cells/well in 100 μL), and the next day the cells were exposed to PP dispersions (200 µg/mL). Cells exposed to cell medium with 1% Triton X−100 (Sigma-Aldrich, USA) served as an LDH positive control. DMEM media containing PP particles and DMEM media without PP-MPs were also tested to check the background influence. After 24 h and 48 h of exposure, the cell medium was removed, the LDH mixture was added (50 μL per well), and the cells were incubated for 30 min in the dark at room temperature. Subsequently, absorbance was measured at 490 nm and 680 nm (reference) using a microplate reader (FLUOstar omega, BMG Labtech, Germany).

#### 2.3.6 Reactive Oxygen Species (ROS) assay

Caco−2 cells were cultured in a black 96-well plate with a cell density of 3 × 10^4^ cells/well in 100 μL culture medium and incubated overnight to ensure attachment to the well bottom. The next day, the culture medium was removed, 100 μL of the PP solution was added (200 µg/mL) and cells were incubated for 24 and 48 h. A ROS positive control group - cells in fully supplemented DMEM media with 10% H_2_O_2_ (Sigma-Aldrich, USA) - and a group of cells with fully supplemented DMEM were incubated simultaneously as PP-treated groups.

To assess intracellular oxidative stress levels, 100 μL of CellROX® Deep Red Reagent was added to the cells, with the final concentration of 5 μM. The cells were then incubated at 37°C for 30 min. Following incubation, the medium was aspirated, and cells were washed three times with PBS 1X. Fluorescence readings were obtained using a fluorescent plate reader (BMG Clariostar, BMG Labtech, Germany) set to excitation/emission wavelengths of 640/665 nm, respectively.

#### 2.3.7 Qualitative analysis of Caco−2 cell culture

**Fig. 1:**
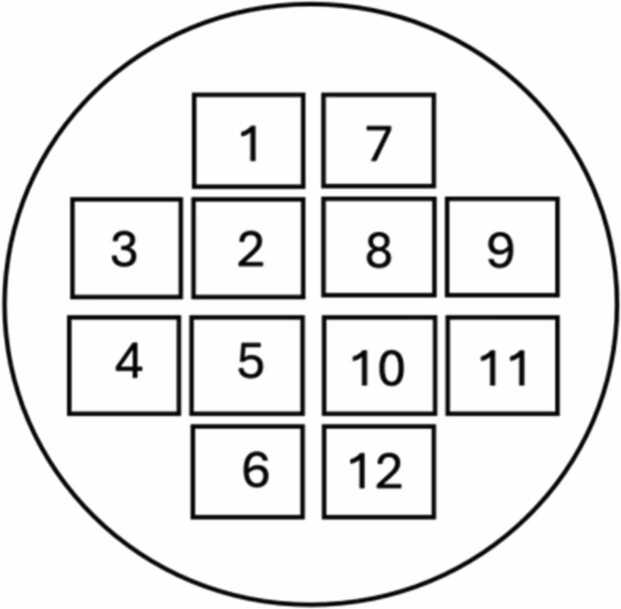
Scheme for image acquisition of Caco−2 cell cultures on a microscope.

Caco−2 cells were seeded at a density of 4 × 10⁴ cells/cm² in culture medium onto clear CellStar, Greiner 6-well culture plates. The cell cultures were imaged after 24 and 48 h using an Olympus CKX53 optical microscope equipped with an Olympus DP23 camera. Digital images were captured at 10 × and 40 × magnifications using CellScan software, with at least 24 images acquired per well to ensure a representative sampling of each sample following the scheme below (Fig. 1). Image preprocessing - adjusting contrast and brightness - was performed using NIH ImageJ software [33].

#### 2.3.8 Statistical analysis

Data are presented as the standard error of the mean (MTT and LDH assays) or fold change (ROS assay) compared to untreated cells (without PP-MPs) and compared to PP Pristine samples (in case of PP-MW samples). At least three independent biological replicates were performed, and each condition was run in triplicate. Statistical analysis was performed using GraphPad Prism Software Version 10.6.1 (San Diego, CA, USA) using one-way (ANOVA) or non-parametric Kruskal-Wallis test with Tukey or Dunnett’s post hoc test for comparisons of multiple groups. Prior to analysis, the normality of all variables was assessed using the Shapiro-Wilk test. Significance levels were set at (p) < 0.05.

## 3 Results and discussion

### 3.1 MP characterisation

#### 3.1.1 Shape, size and electrokinetic properties

**Fig. 2:**
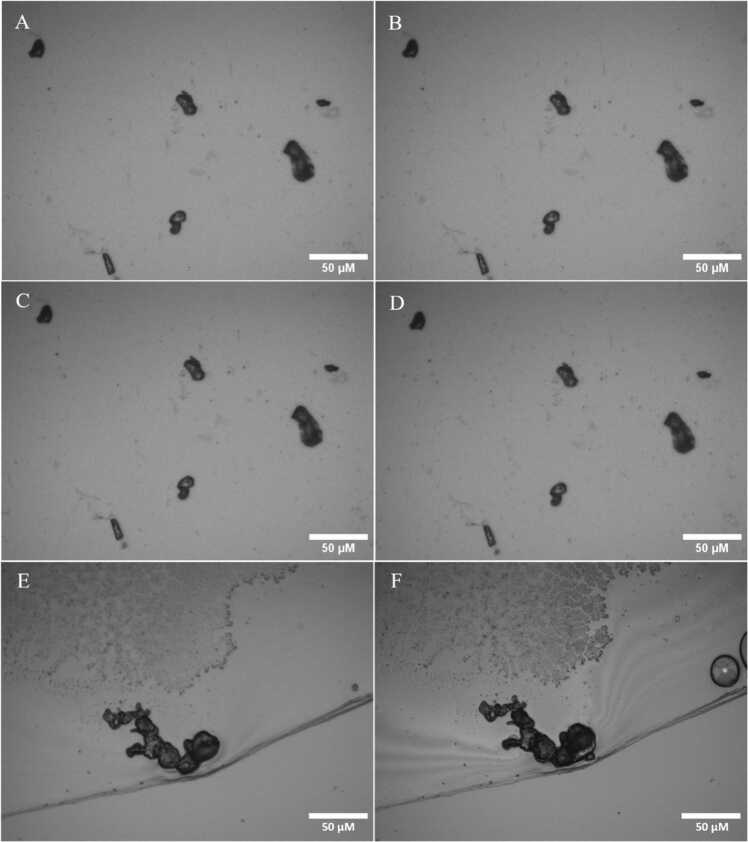
Panels (A-F) illustrate PP MPs before and after MW degradation. Images were acquired using O-PTIR (10 ×). Panel A and E show PP pristine samples; Panels B-D: PP samples following one, five and ten 3-minute cycles, respectively; and Panel F, 30 min of continuous exposure.

Fig. 2 (A–F) illustrates the PP-MPs images acquired using the O-PTIR system. The images reveal that PP-MPs exhibit irregular shapes, consistent with the supplier’s specifications, and their diameters range from 19 to 49 μM (see Table 1).

**Table 1:** Average diameter, circularity and ζ-potential of samples.

**Particles**	**Average diameter (µm)**	**Circularity (%)**	**ζ-potential (mV)**
PP_Pristine	30.69 ± 9.90	77.40	−24.0 ± 4.24
PP_Cycle 1	30.19 ± 10.05	79.40	−26.7 ± 2.00
PP_Cycle 5	30.17 ± 10.01	78.90	−26.6 ± 3.28
PP_Cycle 10	29.48 ± 10.31	79.68	−26.4 ± 1.53
PP_30min	37.14 ± 11.94*	79.44	−33.9 ± 2.71*

*Compared to the pristine samples (PP_Pristine) (p < 0.05).

Table 1 presents the mean particle diameter, circularity and ζ-potential of pristine and MW-treated PP-MPs. All MW-treated samples, except PP_30min, retained particle sizes comparable to the PP_Pristine. In contrast, PP_30min exhibited a statistically significant increase in particle diameter. There were negligible, statistically insignificant changes in the overall particle shape in all MW treatments. It was shown that MW degradation cycles did not affect the general shapes of the PP-MPs. However, the increase in average diameter under prolonged MW irradiation suggests more pronounced morphological alterations, possibly due to localized melting or surface sintering effects [34]. ζ-potential analysis provides information related to the surface charge and colloidal stability of particles (Kwabena [35]). ζ-potential of PP_Pristine was measured at −24.0 ± 4.24 mV, indicating a moderately negative surface charge and the negative ζ-potential value is consistent with previous PP-MPs studies (Kwabena [35], [36]). After one to ten MW cycles, the samples exhibit a slightly more negative potential (Table 1). This modest alteration might result from the formation of hydroxyl groups following thermal treatment [37]. PP_30min showed a more pronounced and statistically significant decrease in ζ-potential (−33.9 ± 2.71 mV), which might point to a higher density of hydroxide groups on the polymer interface by prolonged MW degradation. Moreover, the ζ-potential value indicated that the dispersion of PP_Pristine and one to ten MW cycles was moderately stable, whereas PP_30min dispersion became highly stable due to ζ-potential less than - 30 mV [36]. Shao et al. [17] investigated how variations in zeta potential independently affect cytotoxicity using poly(3-hydroxybutyrate-co−3-hydroxyhexanoate) (PHBHHx) biopolymer particles. Their finding indicated that particles with larger similar charges exhibited increased cytotoxicity to cells. Thus, PP_30min is projected to have higher toxicity to cells than the other samples in our study.

#### 3.1.2 Spectra analysis and PCA

**Fig. 3:**
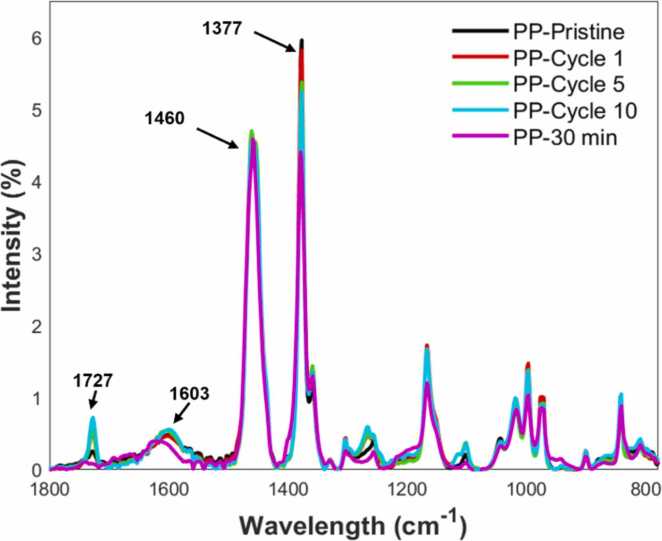
Pre-processed mean spectrum (mid-IR) of PP samples (n ≥ 54).

Spectra of all samples were subjected to pretreatment methods which were moving median, SNV and baseline correction [38] (Fig. 3). The O-PTIR spectra of PP_Pristine exhibit the vibrational signatures of PP polymer, including CH_2_ bending and CH_3_ asymmetric bending around 1460 cm^−1^, CH_3_ symmetric bending near 1377 cm^−1^, and various C-H wagging and rocking vibrations between 840 and 1170 cm^−1^ [39], [40]. In PP_Pristine, there is no discernible absorption in the 1715–1740 cm^−1^ region, indicating negligible oxidative degradation prior to MW degradation [41], [42]. On the other hand, when PP is subjected to repeated MW exposures at 1000 W, the intensity in the carbonyl region progressively increases for PP_Cycle5 and PP_Cycle10, reflecting the formation of oxidation products like ketones, aldehydes or carboxylic acids [2], [37], [41], [42] (Fig. 3). In parallel, the peak at 1377 cm⁻¹ exhibited a significant decrease in intensity with increasing MW cycle number, which might be due to the onset of partial chain scission and localised oxidative reactions [43].

While the weakest carbonyl peak was observed at PP_30min, there was a shift towards larger wavelengths was seen in the 1600–1740 cm^−1^ region, which is associated with oxidative degradation. This discrepancy may arise from advanced oxidative pathways that generate volatile or highly fragmented by-products, inferred from a lower than expected carbonyl peak intensity [44], [45]. Furthermore, the peak intensity of PP_30min is significantly lower than the other samples in the entire mid-IR region (Fig. 3). This decrease might be related to increased particle size as a result of extended MW exposure [46], [47].

**Fig. 4:**
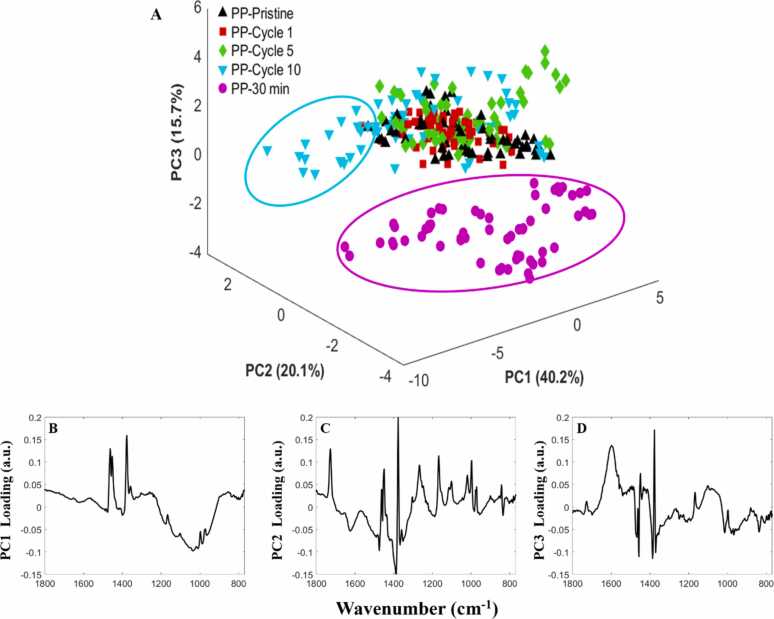
3D PCA score plot (A) and loadings 1 (B), loadings 2 (C), and loadings 3 (D) of PCA.

Score plots of the first three PCs explaining 76% of the variance in the group of IR spectra analysed, are shown in Fig. 4, indicating grouping patterns (Fig. 4 A): PP_Pristine, PP_Cycle1, PP_Cycle5 and PP_Cycle10 occupy overlapping or adjacent regions in 3D space. This proximity indicates that the spectra of MPs exposed to short-cycle treatments (particularly PP_Cycle1 and PP_Cycle5) remain similar to PP_Pristine. On the other hand, PP_Cycle10 indicated a more noticeable shift, which was consistent with a higher carbonyl signal due to oxidation or chain scission. There was a pronounced separation of PP_30min, which clusters far from the other groups along PC2. This distinct positioning suggests that extended MW exposure drives more substantial different chemical modifications.

The PCs spectral loadings highlighted specific wavenumber regions in different ways. PC1 (Fig. 4(B)) was dominated by the CH₂ bend (∼1460 cm⁻¹) and CH₃ symmetric deformation (∼1377 cm⁻¹), together with a clear contribution around 1000 cm⁻¹ assigned to CH rocking characteristic of isotactic PP and sensitive to crystallinity. Although PC1 accounted for the largest share of variance (40.2%), it mainly reflected conformation/crystallinity changes and contributed to within-class dispersion, and only helped tease apart some PP_Cycle10 and PP_30min variability. By contrast, the group separation of PP_30min was driven primarily by PC2 (20.1%), as indicated by the positive PC2 scores of PP_30min versus the negative scores of the remaining samples (Fig. 4(A)). The PC2 loadings (Fig. 4(C)) showed a strong carbonyl feature (1700–1740 cm⁻¹), consistent with oxidation after 30 min MW heating and explaining the separation of PP_30min along PC2. Moreover, the positive peak around 1600 cm^−1^ in PC3 loadings (Fig. 4(D)) might be related to the shifting of the IR spectrum of PP_30min in that region. Overall, the PCA confirms that MW degradation introduces measurable chemical changes to PP-MPs primarily through oxidation and slight alterations in backbone vibrations.

### 3.2 Toxicological cell response

#### 3.2.1 MTT assay

**Fig. 5:**
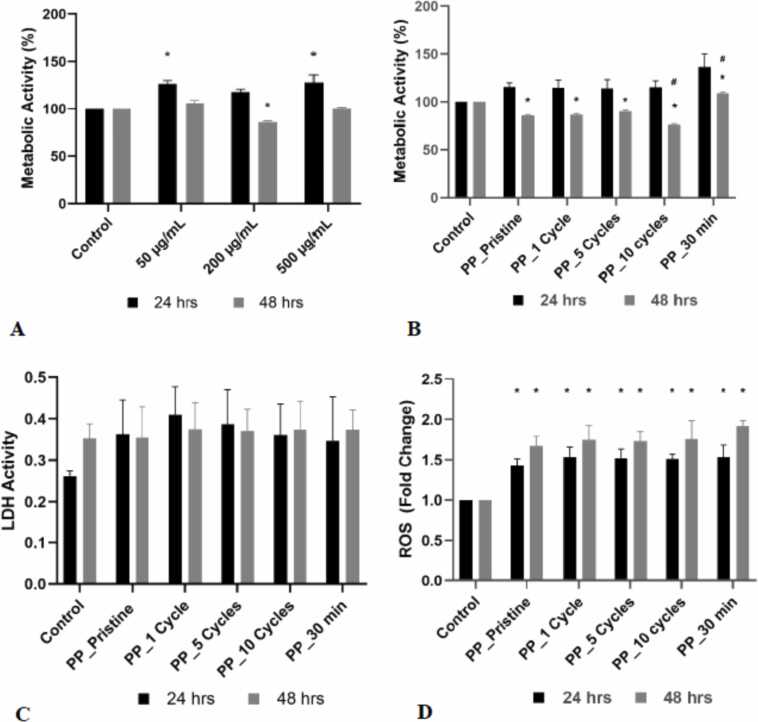
**A-D** shows toxicological assays of Caco−2 cells treated with PP-MPs in different concentrations and MW degradation cycles for 24 and 48 h. Metabolic activity was determined by MTT assay (A-B). Membrane damage was determined by LDH assay (C). Intracellular Reactive Oxygen Species (ROS) was determined by the CellROX® deep red reagent assay (D). Data represent the mean ± SEM of triplicate samples; Asterisk (*) denotes a significant difference between PP-treated groups and untreated group (p < 0.05); Hash (#) denotes a significant difference between PP Pristine and MW groups (p < 0.05).

The 24-hour metabolic activity (Fig. 5A) shows a non-linear response with rates over 100% at 50 μg/mL (126.3 ± 3.8) and 500 μg/mL (127.7 ± 8.4) and a dip at 200 μg/mL (117.7 ± 3.2). Given the PP-MPs properties - large size (∼30 μm) that prevents cellular internalization and a moderately negative zeta potential (−24.0 ± 4.24 mV) that promotes colloidal stability while permitting surface adhesion and protein corona formation - the most parsimonious explanation is that apical particle-cell surface interactions and corona mediated signaling are driving altered metabolic readouts [48], [49]. In undifferentiated, highly proliferative Caco−2 cultures, surface contact, mechanical stimulation, or corona driven modulation of membrane receptors can transiently increase mitochondrial and glycolytic activity [50]. The PP-MPs’ surface chemistry, hydrophobicity, and surface roughness further modulate these effects by shaping the protein corona and adhesive interactions with the apical membrane, changing local signalling and metabolic responses [48], [49].

At 200 μg/mL the particles and their protein coating interact with the cell layer in a way that lowers measured metabolism: intermediate concentrations favour formation of particle aggregates and a distinct protein corona that promote focused, high contact patches on the apical membrane, producing mechanical stress and engaging receptor mediated signaling that transiently down regulates mitochondrial activity or causes detachment of highly metabolic cells, producing the observed dip at 200 μg/mL whereas 50 and 500 μg/mL concentrations yield coronas or particle distributions that provoke compensatory metabolic up regulation in the remaining adherent cells [48], [49], [51].

After 48 h, a return of metabolic readouts at 50 μg/mL (106 ± 3.1) and 500 μg/mL (100.3 ± 1.5) toward control levels is consistent with transient, adaptive cellular responses rather than sustained toxicity: undifferentiated Caco−2 cells can rapidly alter bioenergetic programmes (shift between glycolysis and oxidative phosphorylation), activate repair/adaptive signalling, and resume baseline metabolic rates after an acute perturbation, producing a rebound of assay signals by 48 h. At low concentrations, an early stimulatory effect (temporary up-regulation of metabolic enzymes or proliferation) can dissipate as cells adapt and homeostasis is restored; at high concentrations, initial surface-mediated stimulation or stress can be mitigated over time by changes in particle distribution, corona maturation, or cell reorganization that reduce effective per-cell stimulus, bringing measured metabolic activity closer to untreated levels.

Aggregation, settling, and changes in the particle–protein corona over time also reduce the effective bioavailable particle surface presented to cells [52], so the acute 24 h effects driven by fresh dispersion and high local surface contact are less pronounced by 48 h when clusters have formed or partially sedimented, lowering ongoing mechanical or signalling stimuli. In addition, proliferation of undifferentiated Caco−2 cultures or modest recovery of stressed subpopulations will dilute per cell perturbations, making population level metabolic measures converge on control values. Similar temporal recovery and bioenergetic reprogramming after MPs or NPs exposure have been reported in intestinal cell models.

On the other hand, at 48 h the decline to ∼83% metabolic activity in 200 μg/mL (83.3 ± 1.2) likely reflects delayed, cumulative effects of sustained apical particle contact and concentration dependent corona signalling that shift cells from an adaptive, hypermetabolic state toward reduced mitochondrial function, cell-cycle arrest; sustained mechanical stress from aggregated 30μm PP-MPs and corona mediated engagement of inhibitory receptors can trigger autophagy or apoptosis pathways.

Acute-exposure experiments are essential because they reveal immediate mechanistic responses that often precede longer-erm outcomes and thereby guide targeted follow-up studies [53]. They are also experimentally tractable, enabling standardized workflows and interlaboratory replication so results can be robustly compared and integrated into risk-assessment frameworks [54]. From a toxicological point of view, we selected 200 µg/mL as an upper-bound concentration to ensure robust, reproducible cellular responses and to probe metabolic and/or oxidative pathways that may be undetectable at lower doses. This concentration range choice was made by dose-response prediction model [26], which shows an overall rise in cytotoxic signal with concentration but fluctuations around ∼200 µg/mL - a transition zone where small changes in delivered dose, particle aggregation, or assay sensitivity potentially produce variable readouts.

Fig. 5B illustrates MTT results of Caco−2 cells after exposure to PP-MPs (200 µg/mL) treated with various MW degradation cycles. At 24 h, all samples showed elevated metabolic activity, with PP_Pristine at 116 ± 14.2; PP_Cycle1 at 114.7 ± 8.4; PP_Cycle5 114 ± 9.6;PP_Cycle10 at 115.3 ± 6.9 and PP_30 min continuous at 136 ± 13.7. By 48 h, activity declined in each case, in a cycle-dependent manner: PP_Cycle10 exhibited the lowest metabolic activity rate (76 ± 1.15), while PP_Pristine (85.67 ± 1.2), PP_Cycle1 (86.67 ± 1.2), and PP_Cycle5 (90.3 ± 1.2) decreased by roughly 14%, 13%, and 10%, respectively. This decline suggests that prolonged exposure to PP particles - Pristine and MW-degraded - may impair key cellular functions in longer exposure periods. Although metabolic activity for PP_30 min continuous treatment decreased between 24 and 48 h, it remained upregulated, staying above 100% (109%). In Tukey’s HSD comparisons against PP_Pristine, PP_Cycle10 produced a highly significant metabolic decrease (p < 0.001), and PP_30 min continuous yielded a highly significant metabolic increase (p < 0.001). PP_Cycle5 also caused a modest but significant elevation in metabolic activity (p = 0.044), whereas PP_Cycle1 did not differ significantly (p = 0.67). These findings confirm that prolonged MW degradation (continuous or repeated cycles) drive statistically robust shifts in Caco−2 metabolism relative to PP pristine.

As previously reported in Section 3.1.1. and 3.1.2, when PP-MPs undergo MW degradation they develop oxygenated surface functionalities and release a complex mixture of low molecular weight oxidation products that together reconfigure the protein corona and strengthen particle-cell contact. These chemistry changes (surface oxygenation, altered zeta potential, and release of soluble carbonyls) increase adsorption to the plasma membrane and concentrate reactive species at transporters and receptors, perturbing nutrient influx, ion homeostasis and the local biochemical microenvironment. The downstream consequence is restricted supply of biosynthetic precursors and exacerbated energetic deficits that reduce metabolic throughput and compromise proliferation, biosynthesis and repair [9], [55]. Thermal and MW aging studies of PP-MPs report formation of ketones, aldehydes and carboxylic acids with progressive oxidation, linking the emergence of these oxygenates to surface charge shifts and altered corona composition [9], [56]. Our MW aging data closely match these patterns in both carbonyl speciation and zeta potential shifts, supporting direct relevance of the reported degradation chemistry to the cellular effects observed.

Aldehydes, ketones, and carboxylic acids all originate from related fragmentation pathways but diverge in reactivity, persistence, and cellular impact. Short-chain and α,β-unsaturated aldehydes are highly electrophilic. They rapidly form covalent adducts with nucleophilic residues on enzymes and membrane transporters, diminishing catalytic efficiency and destabilizing metabolic pathways. These changes can trigger rapid declines in glycolytic flux and transporter-mediated uptake [22], [57]. Ketones from PP oxidation are less electrophilic yet more water-soluble and volatile. They desorb from particles, diffuse through the medium, and serve as alternative carbon sources that transiently perturb NAD(P)H pools and elevate dehydrogenase assay signals. Over longer exposures, they act as mild electrophiles to modify protein function [9], [56]. Carboxylic acids remain polar and persistent at the particle–cell interface, where they shift local and intracellular pH. These pH changes impair pH-sensitive enzymes and slow rate-limiting steps in central carbon metabolism, causing buildup of intermediates and an altered redox balance that constrains metabolic flexibility [9], [22].

The differing effects of short versus continuous 30-minute MW treatments arise from their unique physicochemical signatures. Short MW cycles (zeta potential −26 mV) yield particles with persistent, surface-bound oxidation products that strengthen membrane interactions, provoke transporter disruption, foster enzyme adduction, and drive intracellular acidification, collectively suppressing metabolic activity. In contrast, PP_30min treatment (zeta potential −33 mV) generates smaller, volatile fragments and releases ketone-like species; this dual outcome enhances particle–membrane engagement while supplying soluble oxidation products that diffuse into cells or undergo metabolism. These conditions provoke a chemical stress response - upregulating glycolysis and mitochondrial activity - and allow cells to use released oxidation products as auxiliary substrates, together producing the elevated metabolic readouts observed at 24–48 h.

Our findings align with those of Hwang et al. [58], who also observed values above 100% using the CCK−8 assay in human dermal fibroblasts (HDFs) after a 24-hour exposure to PP-MPs suspended in DMSO. In their study, two distinct particle size distributions were investigated - approximately 5–10 μm and 30–50 μm - at concentrations of 10, 50, and 100 μg/mL. The observations exceeding 100% across both methods suggests an acute cellular response characterized by an early compensatory adaptation that is eventually overwhelmed by cytotoxic effects. This pattern is consistent with the biphasic response we observed, highlighting the multifaceted nature of cellular adaptations to PP particle-induced stress.

In contrast, studies such as Xia and Wang [23] have demonstrated cytotoxic effects over similar exposure duration. In their investigation, PP food containers were stored at 4°C for 24 h and then microwaved at 1000 W for 1 min and 40 s, generating PP-MPs with a size distribution ranging from 1 nm to 5 μm. Exposure to these PP-MPs at a concentration of 100 μg/mL resulted in a reduction of cell viability to approximately 70% after 24 h, which further declined to around 65% at 48 h, as measured by the MTT assay. As that study obtained the PP particles from food containers, the additives released from the food containers and the PP size range could be one of the reasons for the difference in toxicity results of PP-MPs to cells when compared to our study. These divergent outcomes, when compared with our results, also draw attention to the role of PP-MP size in modulating cellular health - a finding corroborated by studies from Danopoulos et al. [59], Tang [60], and Kim et al. [61]. To understand more deeply the biological impact of MW-treated PP-MPs and extend the insights obtained from the MTT assay, other endpoints (membrane damage and ROS levels) are explored below.

#### 3.2.2 LDH assay

As depicted in Fig. 5C, LDH activity in the MW-treated samples was generally comparable to the PP_Pristine, with the exception of a slightly elevated LDH signal in PP_Cycle 1 after 24 h of exposure. Although this increase did not reach statistical significance at either 24 or 48 h, the data suggests that PP exposure – Pristine and MW degraded - might cause minor perturbations to cell membrane. It is also important to consider that the observed variability may stem from technical factors such as assay sensitivity [62], particle aggregation or inconsistent particle distribution [63], underscoring the potential need for complementary assays to fully capture the spectrum of cell membrane responses after MP exposure. Importantly, our measurements confirmed that MW degradation yields PP-MPs with a more negative ζ-potential (Table 1). This finding supports the hypothesis that MW exposure modifies the particle surface characteristics, which may enhance adhesion to cell membranes. Previous studies have shown that MPs with a more negative ζ-potential tend to adhere more strongly to cell surfaces, potentially facilitating greater cellular interaction and contributing to cytotoxic effects [64], [65]. In addition, the slight increase in LDH at 24 h may also reflect localized mechanical stress resulting from particle deposition on the cell surface, as suggested by Fleury and Baulin, [66]. However, even with this measured shift in ζ-potential, the modest LDH elevation remains at a low level, and the mechanistic link to membrane disturbance cannot be definitively established yet. Future studies employing high-resolution imaging or alternative markers of membrane integrity may help clarify this relationship.

Comparative analysis further emphasizes the need for standardized methodologies [60]. For instance, Rousgaard et al. [67] reported no significant LDH changes in Caco−2 cells exposed to PP debris with particle sizes ranging from 80 to 250 nm and 200–600 nm, whereas Jin et al. [7] documented a substantial increase in LDH release in cells exposed to resuspended PP microparticles (average size 396.4 nm) after MW degradation. Moreover, discrepancies in particle size measurement - such as the supplier’s reported range of 55–75 µm versus our measured average of 30.69 ± 9.90 µm - point out the urgent need for improved MP characterisation techniques. Standardising both the physical characterisation and biological assay protocols will be crucial in resolving these inconsistencies and establishing a clearer understanding of how particle properties influence cytotoxic outcomes.

Due to the relatively large size (average 30.7 µm; see Table 1), the PP-MPs used in our study are less likely to be internalized by epithelial cells [58], such as Caco−2; instead, they tend to adhere to the cell surface, where they can induce local mechanical stress and potentially compromise membrane integrity [68]. Furthermore, the moderately negative ζ-potential (–24.0 ± 4.24 mV; see Table 1) of PP_Pristine not only ensures a stable dispersion in aqueous media but also promotes the formation of a protein corona as serum proteins and other biomolecules adsorb onto the particle surface [69]. This protein corona can modify the effective surface properties of the PP particles, influencing their interactions with cellular membranes and facilitating the activation of signalling pathways that lead to ROS generation [70], [71].

#### 3.2.3 Reactive oxygen species (ROS) assay

Oxidative stress occurs when there is an imbalance between the production of ROS and the cellular antioxidant mechanisms responsible for their detoxification and neutralization [72]. Like all other cell types, enterocytes produce reactive oxygen species as a result of normal cell metabolism and need to be equipped with the different proteins involved in oxidant removal and damage repair [73].

The intracellular ROS analysis, as depicted in Fig. 5D, shows a consistent and statistically significant increase in ROS levels following exposure to PP-MPs under various MW-degraded conditions. At 24 h, PP_Pristine induced a 1.426-fold increase in ROS, whereas PP_Cycle1 yielded a 1.537-fold rise, PP_Cycle5 1.524-fold, PP_Cycle10 1.513-fold, and PP_30min 1.534-fold. By 48 h, ROS accumulation had intensified: PP_Pristine reached 1.675-fold, PP_Cycle1 1.749-fold, PP_Cycle5 1.731-fold, PP_Cycle10 1.757-fold, and PP_30min produced the highest level at 1.918-fold. These data show that although all MW degradation treatments elevate oxidative stress over time, PP_30min drives the most pronounced ROS response at the later time point.

The concept of MPs as a key driver of oxidative stress has garnered considerable attention and it has been reported in different *in vitro* biosystems such as polystyrene (PS) in HDFs [74]; and polyethylene (PE) in T98G cells [75]. In terms of PP-MNPs studies, oxidative stress has been reported in several cell lines such as H9C2 [76], A549 [77] and differentiated THP−1 macrophages [78]. In studies utilizing Caco−2 cells, Hwang et al. [58] observed that exposure to mechanically degraded PP-MPs - with an average size of approximately 25 μm, suspended in DMSO at a concentration of 1000 μg/mL - resulted in a 30% increase in ROS levels after 24 h. In a related investigation, Jin et al. [7] evaluated the effects of resuspended PP nanoparticles (approximately 396.4 nm) derived from food containers subjected to MW degradation (30 s at 700 W). Following 48 h of exposure, ROS levels in Caco−2 cells were elevated by approximately 1.5-fold relative to control conditions. Collectively, these findings emphasize the significant role of oxidative stress as a mediator of cytotoxicity induced by environmentally degraded PP, highlighting how both particle size and degradation method contribute to adverse cellular outcomes.

Literature indicates that MPs can act as inducers of ROS due to their intrinsic properties [72], [79]. For instance, the MPs high surface area-to-volume ratio facilitates direct interactions with cellular membranes and the adsorption of serum proteins, potentially perturbing membrane integrity and triggering intracellular signalling cascades that culminate in ROS production [65], [80]. In the context of our study, under MW irradiation, PP-MPs are subjected to localized thermal stress, which elevates the kinetic energy of their molecular bonds. This increased energy predisposes the polymer chains to homolytic cleavage of C–H and C–C bonds (Fig. 3), generating free radicals that initiate chain reactions, leading to polymer degradation [65], [81], [82]. As described in Section 3.1.2, MW degradation introduces oxygen-containing functional groups (e.g., carbonyl, hydroxyl) into the PP structure (Fig. 3), which might reduce its molecular weight and produce more hydrophilic surfaces, according to [63]. As shown in 3.2.1, 3.2.2, the zeta potential results (Table 1) correlate with changes in intracellular ROS. The progressive increase in negative zeta potential likely strengthens electrostatic interactions with the cell membrane, promoting membrane perturbation and amplifying oxidative stress in a degradation- and time-dependent manner. These surface changes therefore increase particle–membrane interactions and disrupt normal cellular functions, including elevated intracellular ROS levels.

**Fig. 6:**
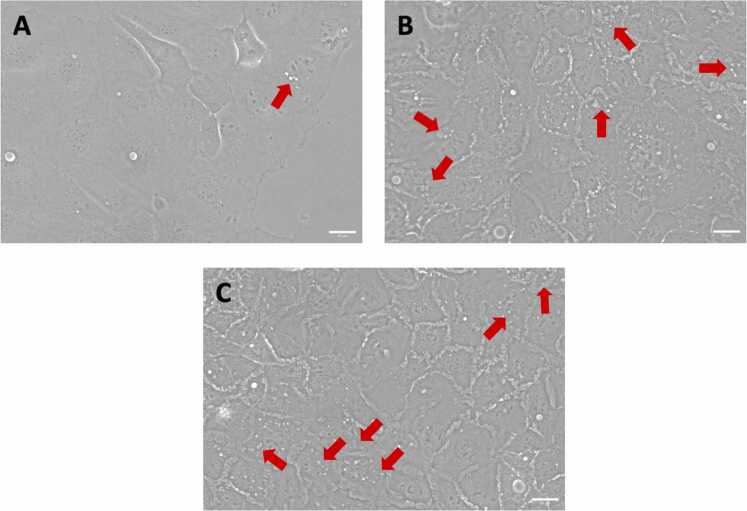
**A-C** show representative fields illustrating morphology of Caco−2 cells 48 h exposure to PP-MW-MPs. A) Control; B) PP_Pristine; C) PP_30min. Magnification 40x; Size bar 20 µm.

Although intracellular ROS was elevated across all PP treatments (Fig. 6C), spectral analysis and PCA (Fig. 3 and 4, respectively) show that PP_Cycle10 and PP_30min produced the most pronounced chemical alterations. PP_Cycle10 markedly suppressed Caco−2 metabolism relative to PP_Pristine and induced a 1.7-fold increase in ROS compared with untreated cells, suggesting oxidative-stress–mediated disruption of redox homeostasis. By contrast, PP_30min triggered a hypermetabolic response accompanied by a 1.9-fold rise in ROS. These opposing metabolic effects are consistent with the distinct suites of degradation products generated by each MW treatment and emphasize the central role of polymer breakdown chemistry in driving cellular responses.

After 10 MW cycles (PP_Cycle10), the presence of three oxidation products was indicated: aldehydes, ketones and carboxylic acids. Aldehydes, due of their high reactivity, increase the ROS levels in Caco−2 cells through glutathione depletion and mitochondria damage, causing electron leakage [83] and by triggering Ca²⁺ influx that activates NADPH oxidase [84]. Ketones are a naturally produced metabolite and have diverse roles in the regulation of cellular processes such as metabolism, inflammation, and cellular crosstalk [85]. However, in other cell lines the effect of excessive ketones exposure has been reported: in cultured neurons, cardiomyocytes and microvascular endothelial cells, respectively, exposure to supraphysiological ketone levels elevated ROS, driving moderate oxidative stress beyond the protective effects seen at physiological concentrations [85], [86], [87]. Finally, the build-up of carboxylic acids may lower the local pH, compromising key enzymatic activities and disrupting cellular homeostasis [88]. This acidification, combined with the propensity of carboxylic acids to further enhance ROS generation [89], likely exacerbates the oxidative damage initiated by the degradation products described above.

On the other hand, chemical modifications unique to PP_30min indicate that advanced oxidative pathways - leading to the generation of volatile or highly fragmented by-products – potentially act as a key mechanism driving the observed increase in ROS [90]. The oxygenated moieties in these by-products can initiate lipid peroxidation via hydrogen abstraction, subsequently propagating Fenton and Haber–Weiss reactions that generate ROS such as superoxide anions, hydrogen peroxide, and hydroxyl radicals [70], [72]. Moreover, these by-products may disrupt mitochondrial electron transport - impairing oxidative phosphorylation and causing electron leakage that further increases ROS production. They are also capable of activating cell surface receptors and intracellular signalling cascades, including those mediated by NADPH oxidase, NF-κB, and MAPK, thereby amplifying inflammatory responses [22], [91].

Our study indicates that the inherent properties of PP-MPs, combined with MW degradation and prolonged exposure, overwhelm the antioxidant defences of Caco−2 cells, rendering them unable to counteract the resulting excess ROS production. The elevated oxidative stress levels would initially stimulate cellular metabolic activity by mobilizing defence mechanisms and activating survival pathways, with ROS acting as signalling molecules [91], [92]. This activated state may account for the temporary increase in metabolic activity results. As cells attempt to counteract the oxidative stress and maintain homeostasis, disruptions in metabolic pathways may occur, potentially affecting nutrient absorption and overall cell function, as previously reported in Fig. 6B. These adaptive responses indicate the dynamic between oxidative stress and cellular metabolic activity, highlighting the central role of ROS in modulating cellular outcomes.

#### 3.2.4 Effect of Microwave Degradation on Cell Morphology

In addition to quantitative measures obtained from the cytotoxicity assays, cell morphology was examined to provide qualitative insights into the cytotoxic effects induced by PP exposure. The panel below (Fig. 6) shows images of Caco−2 cells before and after exposure to MW treated PP-MPs.

After 48 h of treatment, Caco−2 cells exposed to both pristine and MW-degraded PP-MPs exhibited a marked increase in intracellular vacuolization (Fig. 6, red arrows). The degree of vacuole formation was most pronounced in PP_30min. While vacuole formation is a recognized morphological response in Caco−2 cells [93], PP-MPs exposure significantly amplified this phenomenon after 48 h [94]. Importantly, extensive intracellular vacuolization has been correlated with the early stages of apoptosis [95], [96].

The presence of vacuoles is linked with the sustained generation of ROS previously reported (Fig. 5D) as it underlies many apoptotic cascades: prolonged oxidative stress compromises mitochondrial integrity, triggers cytochrome c release, and activates downstream caspases [97], [98], [99]. In line with this mechanism, Lu et al. [76] reported that PP-MPs (2–5 µm) induce cardiomyocyte apoptosis via ROS-mediated activation of the MAPK–Nrf2 signalling axis, ultimately promoting myocardial fibrosis and functional decline. Although their study employed a smaller particle size range, it reinforces the concept that PP-derived particles provoke oxidative stress and apoptotic pathways in mammalian cells

Based on the information obtained from the toxicological assays, the morphological analysis and understanding that the intestinal epithelium undergoes a continual process of proliferation, differentiation and apoptosis, we suggest that Caco−2 cells exposed to PP-MPs experienced metabolic overstimulation due to excessive ROS production and the cell cycle was shortened, leading to an early apoptosis stage after 48 h.

### 3.3 Limitations, future directions and study significance

The present study has certain limitations that should be acknowledged. First, the use of a single‑cell culture model rather than a co‑culture system restricts our ability to mimic the complexity of the intestinal environment, including interactions between epithelial, immune and goblet cells. Second, the PP MPs used here contained no food‑contact additives such as antioxidants or UV stabilizers, which may influence degradation pathways and physicochemical behaviour in real‑world scenarios. Third, the spectral analysis was limited to 769–1800 nm (near‑infrared), a region dominated by broad overtone and combination bands that indicate general functional groups (e.g., increased C–H, O–H or carbonyl‑type signals) but lack the sharp, diagnostic peaks required to identify specific oxidation products or structural isomers. As a result, this window can reveal increased oxidation and broad chemical classes such as ketones or aldehydes, but cannot confirm exact molecular identities, quantify trace species reliably, or resolve closely related compounds. Finally, we acknowledge that translating these *in‑vitro* findings to real‑world exposure requires lower‑dose testing or the application of physiologically based dosimetry models. Future research should focus on several directions to enhance our understanding of PP-MPs and their effects on intestinal cells. Studies utilizing environmental PP samples and investigating the behaviour of PP-NPs are crucial to provide a comprehensive and realistic assessment of their impacts. Exploring co-culture systems to mimic the structure and function of the small intestine could improve the accuracy of *in vitro* models. Investigations should explore the dynamic responses of individual cell organelles during PP-MPs exposure, particularly to identify potential disruptions in organelle interactions. Understanding these disruptions may shed light on the mechanisms behind increased ROS levels and oxidative stress, necessitating further research to pinpoint the primary sources of ROS generation and the specific pathways involved. Finally, follow up with targeted GC‑MS (gas chromatography–mass spectrometry) and LC‑MS (liquid chromatography–mass spectrometry) analyses to identify and quantify aldehydes, ketones and carboxylic acids and link their temporal release to observed biological effects. These approaches will pave the way for establishing a more accurate framework for assessing the risks associated with MP exposure.

The study reinforces the importance of MP characterisation, which is essential not only for understanding their physicochemical properties but also for predicting their fate, transport, and interactions with diverse matrices, such as cells and tissues. Moving forward, further research should explore the influence of varying environmental conditions on PP-MPs and broader implications for human health, aiming to establish safer practices for food storage and consumption.

## 4 Conclusions

This study demonstrates that MW degradation significantly alters the physicochemical characteristics of PP-MPs, which in turn influence their biological interactions with Caco−2 cells. Characterisation of the PP particles using O-PTIR revealed changes in parameters such as size, spectra and ζ-potential after MW degradation. Furthermore, PCA analysis confirmed the chemical differences among the MW-treated samples, particularly the distinct separation observed in PP_30min, suggesting that these modifications may enhance the particles’ propensity to interact with cellular membranes. PP characterisation not only supports the potential for MW-induced degradation but also lays the groundwork for a more in-depth discussion of the underlying thermal/oxidative mechanisms driven by these chemical changes and their consequences to Caco−2 cells exposure. Our toxicological assays showed a complex, time-dependent cellular response. An initial increase in metabolic activity at 24 h - possibly an adaptive response - was followed by a decrease at 48 h, indicating that prolonged exposure to modified PP particles leads to accumulated cellular stress and functional impairment. Complementary LDH assays further corroborated these findings by revealing modest indications of localized membrane damage under all MW conditions, while the intracellular oxidative stress measurement reveal a significant increase of ROS over time. On the other hand, PP_30min showed a persistent upregulated metabolic activity and ROS levels after 48 h when compared to shorter MW cycles, reinforcing the different chemical products formed during MW cycles affected directly the cell response. Observing morphological aspects, after 48 h, Caco−2 cells treated with both pristine and MW-degraded PP-MPs showed a cycle-dependent surge in intracellular vacuolization - most pronounced PP_30min - a response that parallels sustained ROS generation and suggests early apoptotic signalling. Our study holds significant relevance as it mimics the everyday domestic use of microwaves, emphasizing the importance of research designed to replicate real-world environmental conditions. It draws attention to the potential health consequences associated with the widespread use of plastic containers for food storage and consumption, highlighting how routine practices can inadvertently lead to exposure to PP-MPs.

## CRediT authorship contribution statement

**Jun-Li Xu:** Writing – review & editing, Validation, Supervision, Resources, Project administration, Investigation, Funding acquisition, Data curation, Conceptualization. **Emine Merve Canga:** Writing – review & editing, Writing – original draft, Methodology, Investigation, Formal analysis, Data curation. **Raphaela O.G. Ferreira:** Writing – review & editing, Writing – original draft, Methodology, Investigation, Formal analysis, Data curation, Conceptualization. **Tara McMorrow:** Writing – review & editing, Validation. **Aoife Gowen:** Writing – review & editing, Validation, Supervision.

## Declaration of Competing Interest

The authors declare the following financial interests/personal relationships which may be considered as potential competing interests: Jun-Li Xu reports financial support was provided by Scientific Foundation Ireland. If there are other authors, they declare that they have no known competing financial interests or personal relationships that could have appeared to influence the work reported in this paper.

## Data Availability

Data will be made available on request.
